# Influence of Grain-Scale Heterogeneity on Hydraulic Fracturing: A Study Based on a Hydro-Mechanical Phase-Field Model

**DOI:** 10.3390/ma19071322

**Published:** 2026-03-26

**Authors:** Gen Zhang, Cheng Zhao, Zejun Tian, Jinquan Xing, Jialun Niu, Zhaosen Wang, Wenkang Yu

**Affiliations:** 1 College of Engineering, Xizang University, Lhasa 850000, China; zroot@utibet.edu.cn (G.Z.); wzs15539969235@163.com (Z.W.); 17888043600@163.com (W.Y.); 2 Xizang Autonomous Region Plateau Major Infrastructure Intelligent Construction and Resilient Safety Technology Innovation Center, Xizang University, Lhasa 850000, China; xingjq@tongji.edu.cn (J.X.); niujialun@tongji.edu.cn (J.N.); 3 Department of Geotechnical Engineering, Tongji University, Shanghai 200092, China; 2431014@tongji.edu.cn

**Keywords:** hydraulic fracturing, phase field method, crystalline rock, grain size, mineral distribution

## Abstract

Heterogeneity at the grain scale strongly influences hydraulic fracturing in crystalline rock; however, systematic studies quantifying its impacts on the evolution of injection pressure and crack propagation remain limited. To address this gap, we employ a hydro-mechanical phase-field model incorporating Voronoi-based microstructures to systematically quantify the effects of grain-scale heterogeneity on hydraulic fracturing. Two numerical experimental programs are designed to examine the effects of (i) mean grain size and (ii) mineral distribution under different axial stresses. The simulations reveal a close coupling between injection pressure and crack-length evolution, and both responses are strongly governed by grain-scale heterogeneity. When the fracture enters weak minerals, it advances rapidly and pressure drops; when it encounters on strong minerals, growth slows or arrests and pressure builds until a threshold triggers the next advance. Moreover, peak pressure statistics further indicate that mineral distribution dominates the response scatter, while axial stress plays a secondary role. Specifically, the mean peak pressures at 0 and 10 MPa are similar (about 14.31 and 14.21 MPa), whereas rearranging minerals within the same Voronoi tessellation changes peak pressure by more than 4 MPa. Higher peaks occur when strong minerals lie ahead of the initial crack tip, increasing resistance to initiation and early growth. Finally, the stress state modulates fracture trajectories: under low axial stress, fractures preferentially follow mineral boundaries, whereas higher axial stress strengthens macroscopic stress guidance and shifts the path toward a direction closer to being perpendicular to the maximum principal stress. This trend is consistent with energy minimization, since interface detouring under high axial stress incurs a larger elastic free energy penalty.

## 1. Introduction

Hydraulic fracturing is a pivotal technique in geotechnical and petroleum engineering. The mechanics of fluid-driven fracture growth have been extensively studied from both fundamental and applied perspectives [1,2,3]. Despite rapid progress, field observations and laboratory evidence consistently indicate that fracture trajectories and breakdown responses deviate markedly from idealized predictions, particularly in heterogeneous rocks [4,5]. This discrepancy underscores that the influence of rock heterogeneity on hydraulic fracture propagation remains insufficiently understood. Consequently, investigating hydraulic fracturing at the grain scale is essential to clarify the mechanisms by which microstructural heterogeneity governs fracture-path evolution and macroscopic failure behavior.

With continued advances in loading systems, sensing, and imaging, laboratory hydraulic-fracturing experiments are increasingly used to investigate fracture initiation, path development, and breakdown behavior in heterogeneous rocks. Acoustic emission (AE) provides real-time localization of microcracking and event statistics that reflect damage evolution under varying stress states and injection schedules [6,7]. At the mesoscale, non-destructive X-ray CT enables 3D reconstruction and quantitative descriptors of fracture morphology, while recent workflows explicitly combine AE with CT to link temporal damage sequencing to volumetric fracture visualization [8,9]. Full-field optical methods such as digital image correlation (DIC), often integrated with high-speed imaging, capture surface strain localization and fracture process-zone growth, offering direct links between heterogeneity-controlled deformation and macroscopic breakdown behavior [10,11,12]. However, experimental methodologies pose inherent challenges in strictly controlling specific research variables and isolating the effects of confounding factors, leading to considerable variability in results.

Numerical simulation has become indispensable, offering precise and independent control over variables compared to experimental methods. Commonly used numerical approaches for hydraulic fracturing include the Finite Element Method (FEM), the extended Finite Element Method (XFEM), the Displacement Discontinuity Method (DDM), and the Discrete Element Method (DEM). FEM typically represents fracture opening and propagation via cohesive-zone modeling (CZM) or interface elements, providing a straightforward framework for fluid–solid coupling at the fracture surfaces and thus being widely used in engineering-scale analyses [13,14]. XFEM extends the standard FEM formulation by introducing enrichment functions that allow displacement discontinuities to be captured within elements, thereby reducing the need for frequent mesh regeneration and improving robustness for crack-growth simulations [15,16,17]. DDM discretizes the fracture surface using displacement discontinuity elements, and its fracture-surface-only discretization makes it computationally efficient for problems dominated by a limited number of fractures [18,19]. DEM, in contrast, describes fracture initiation and network evolution as an emergent result of particle/bond interactions, which makes it well suited for representing heterogeneous media and complex fracture networks [20,21,22]. In the modeling of rock heterogeneity, the Grain-Based Model (GBM) is widely used to characterize the microstructure of such materials. Originally developed by Potyondy [23] within the Particle Flow Code (PFC) 2D, GBM represents rock microstructure using polygonal particles composed of multiple small disks. This method allows for precise control over grain types and their size distributions, contributing to its extension to other numerical approaches and broad application [24,25,26].

While traditional crack propagation simulation methods are widely used, they typically require explicit fracture geometry representation and sophisticated path-tracking algorithms, making it difficult to handle complex fracture behaviors. By contrast, the phase-field method (PFM), which eliminates the need for explicit crack tracking and enables the capture of complex fracture behaviors such as crack merging, branching, non-planar propagation, and three-dimensional crack growth [27,28], has therefore attracted substantial attention in recent years [29,30,31]. The phase field method is grounded in the variational principle proposed by Francfort and Marigo [32], which postulates that the physically realized displacement field and crack set are those that minimize the total energy of the system. In the phase-field framework, damage is represented by a continuous scalar field, thereby regularizing discrete crack surfaces [33]. Wu [34] established a unified phase-field model by combining Barenblatt cohesive zone model and Griffith brittle fracture theory, which alleviates the dependence of simulations on the internal length scale parameter [35]. This framework also offers a new avenue for investigating grain-scale rock fracturing problems. The phase field model has been extensively applied to hydraulic fracturing research and has been systematically reviewed by scholars [36,37,38]. Building on the unified phase field framework, we previously developed a segregated hydro-mechanical solution scheme that yields global responses nearly independent of the length scale parameter [39,40]. These developments underscore the capability and potential of phase-field models for predicting hydro-mechanical fracturing, forming the basis of this study.

Building on our previously developed hydro-mechanical phase-field model, we conduct a controlled, systematic investigation of grain-scale heterogeneity effects on hydraulic fracturing in crystalline rock. We designed two numerical experimental programs to specifically examine the impacts of (i) mean grain size and (ii) mineral distribution under varying axial stress levels, employing constant-rate injection into a pre-existing crack. By analyzing hydraulic fracture growth and its associated injection pressure response, we clarify the dominant role of heterogeneity, with axial stress providing a secondary modulation of fracture path selection. Ultimately, these findings bridge the gap between mesoscopic heterogeneity and macroscopic fracturing responses, offering new insights into the fundamental mechanisms governing fluid-driven failure in heterogeneous rocks.

## 2. Introduction of Hydro-Mechanical Phase-Field Model

### 2.1. Phase Field Model of Fracture in Poroelastic Media

In the phase field method, a scalar field d is introduced to regularize cracks, and the degree of crack smearing is governed by the phase field length scale parameter $l_{0}$. This scalar field, referred to as the phase field, takes values from 0 to 1, representing the transition of the material from intact to fully fractured.

To characterize the degradation of the material’s ability to store strain energy with increasing damage, an energy degradation function ω(d) is introduced; it ranges from 0 to 1 and monotonically decreases. The effective strain energy $\Psi_{\epsilon }$ stored in the damaged material is given by:

(1) $$\Psi_{\epsilon }=\int_{\Omega }\omega (d)\psi_{0}(\epsilon )d\Omega ,\;\psi_{0}(\epsilon )=\frac{1}{2}\epsilon :E_{0}:\epsilon$$

Here, $\psi_{0}(\epsilon )$ denotes the elastic energy density of the intact material. In this study, the degradation function is defined as:

(2) $$\omega (d)=\frac{{\left(1-d\right)}^{2}}{{\left(1-d\right)}^{2}+a_{1}d\cdot (1-\frac{1}{2}d)}$$

Here, $a_{1}=\frac{4}{\pi l_{0}}\frac{E_{0}G_{c}}{\sigma_{c}^{2}}$, $E_{0}$ is the elastic modulus, $G_{c}$ is the surface energy density, and $\sigma_{c}$ is the tensile strength.

Compared with the degradation function used in classical phase-field models, the above formulation (2) ensures that numerical results within the unified phase field framework are insensitive to the internal length parameter, and that crack nucleation occurs only when the critical stress $\sigma_{c}$ is reached, which is more consistent with the actual failure behavior of rock materials.

Crack evolution is driven by the system’s total energy functional, and the system evolves toward a configuration that minimizes the total energy over all admissible states. Accordingly, the governing equations of the phase-field fracture model are derived from the first variation in the total energy functional Ψ, which is written as:

(3) $$\delta \Psi =\frac{\partial \Psi }{\partial u}:\delta u+\frac{\partial \Psi }{\partial d}:\delta d+\frac{\partial \Psi }{\partial \nabla d}:\delta \nabla d=0$$

In poroelastic media, the total energy functional Ψ of the system can be expressed as the sum of the fracture energy $\Psi_{f}$, the elastic energy $\Psi_{\epsilon }$, the fluid pressure-related term $\Psi_{p}$, and the external work $W_{ext}$.

According to the phase field approach, the fracture energy $\Psi_{f}$ can be expressed as:

(4) $$\Psi_{f}=\int_{\Gamma }G_{c}dS=\int_{\Omega }G_{c}\gamma (d,\nabla d)d\Omega$$

where γ(d,∇d) is the crack surface density functional. According to the unified phase field theory proposed by Wu [34], it is given by:

(5) $$\gamma (d,\nabla d)=\frac{1}{\pi }(\frac{1}{l_{0}}(2d-d^{2})+l_{0}|\nabla d{|}^{2})$$

To characterize the work done by the fluid pressure p on the deformation of the porous skeleton, the potential $\Psi_{p}$ is written as

(6) $$\Psi_{p}=\int_{\Omega }\alpha p\cdot (\nabla \cdot u)d\Omega$$

where α is the Biot coefficient. The external work is given by:

(7) $$W_{\mathrm{ext}}=\int_{\Omega }b\cdot ud\Omega +\int_{\partial \Omega_{n_{i}}}f\cdot udS$$

which describes the work done by the body force b and the traction f on the Neumann boundary.

By taking variations with respect to the displacement field and the phase field, the strong-form governing equations can be obtained as:

(8) $$\left\{\begin{array}{l} \nabla \cdot \sigma^{por}+b=0\\ -\omega^{\prime }(d)\psi_{0}(\epsilon )=-\omega^{\prime }(d)y=\frac{2G_{c}}{\pi }(l_{0}\nabla^{2}d-\frac{1-d}{l_{0}}) \end{array}\right.$$

Here, $\sigma^{por}=\sigma -\alpha pI$.

However, $y=-\psi_{0}(\epsilon )$ is symmetric with respect to tension and compression. For hydraulic fracturing dominated by tensile failure, it can be modified using the maximum-stress criterion as:

(9) $$\bar{y}=\frac{1}{2E_{0}}{\langle {\bar{\sigma }}_{1}\rangle }^{2}$$

Here, ${\bar{\sigma }}_{1}=\max(0,\frac{\sigma_{1}}{\omega (d)})$, which ensures that the maximum principal effective stress is set to zero under compression.

Considering the irreversibility of material damage, the reference energy must evolve monotonically. Therefore, a local history field variable H is introduced, which takes the maximum value of $\bar{y}$ during the evolution of the system over s∈[0,t]:

(10) $$H=\max_{s\in [0,t]}(\bar{y})=\max_{s\in [0,t]}(\frac{1}{2E_{0}}{\langle {\bar{\sigma }}_{1}\rangle }^{2})\;\mathrm{in}\;\Omega \times [0,T]$$

Finally, the governing equations can be expressed as:

(11) $$\left\{\begin{array}{l} \nabla \cdot \sigma^{por}+b=0\\ -\omega^{\prime }(d)H=\frac{2G_{c}}{\pi }(\frac{1-d}{l_{0}}-l_{0}\nabla^{2}d) \end{array}\right.$$

### 2.2. Hydro-Mechanical Coupling Equations

As the degree of material damage evolves, the flow characteristics of the poroelastic medium also change accordingly. To represent the relationship between flow parameters and the phase field, the indicator-function approach proposed by Lee et al. [41] is adopted in this study. This method employs two thresholds $\phi_{c1}$ and $\phi_{c2}$ to partition the porous medium into a reservoir domain $\Omega_{R}$, a transition domain $\Omega_{T}$, and a fractured domain $\Omega_{F}$. Two linearly interpolated indicator functions are used to describe fluid parameters affected by the phase field:

(12) $$\chi_{R}=\left\{\begin{array}{ll} 1 & \phi \in [0,\phi_{c1})\\ \frac{\phi_{c2}-\phi }{\phi_{c2}-\phi_{c1}} & \phi \in [\phi_{c1},\phi_{c2})\\ 0 & \phi \in [\phi_{c2},1] \end{array}\right.\;,\;\chi_{F}=1-\chi_{R}$$

Accordingly, the porosity $\epsilon_{p}$, Biot coefficient α, and permeability K can be expressed as:

(13) $$\epsilon_{p}=\chi_{R}\epsilon_{pR}+\chi_{F}\epsilon_{pF},\;\alpha =\chi_{R}\alpha_{R}+\chi_{F}\alpha_{F},\;K=\chi_{R}K_{R}+\chi_{F}K_{F}$$

Here, subscripts *R* and *F* denote the corresponding parameters in $\Omega_{R}$ and $\Omega_{F}$, respectively. The fractured domain is assumed to be fully filled with fluid; therefore, $\epsilon_{pF}=1$ and $\alpha_{F}=1$. Other fluid properties, such as fluid density $\rho_{f}$, dynamic viscosity μ, and fluid compressibility c, are not affected by the phase field.

In this study, the governing equation for fluid flow is derived using Darcy flow and the storage model within quasi-static Biot theory [42]. In a saturated quasi-static Biot system, pores are fully filled with fluid; hence, the increment of fluid content can be written as:

(14) $$\rho_{f}\frac{\partial \zeta }{\partial t}=\rho_{f}\frac{\partial \epsilon_{p}}{\partial t}+\rho_{f}\alpha \frac{\partial \epsilon_{vol}}{\partial t}$$

Here, $\rho_{f}$ is the fluid density and $\epsilon_{vol}=\epsilon_{ii}$ denotes the volumetric strain. Within the phase-field framework, the regularized crack opening is equivalent to the large deformation in the fully fractured region; thus, in the fractured domain, the volumetric strain physically represents the change in storage space available for fluid.

By introducing a source term $Q_{m}$ accounting for fluid transport and external exchange, the fluid mass conservation equation is:

(15) $$\frac{\partial }{\partial t}(\epsilon_{p}\rho_{f})+\nabla \cdot (\rho_{f}v)=Q_{m}-\rho_{f}\alpha \frac{\partial \epsilon_{vol}}{\partial t}$$

where v is the fluid velocity. According to the storage model, the first term on the left-hand side in Equation (15) can be expressed as:

(16) $$\frac{\partial }{\partial t}(\epsilon_{p}\rho_{f})=\rho_{f}S\frac{\partial p}{\partial t}$$

In this equation, S is the storage coefficient, defined as:

(17) $$S=\epsilon_{p}c+\frac{\left(\alpha -\epsilon_{p})(1-\alpha \right)}{K_{V}}$$

and $K_{V}=\frac{E_{0}}{3(1-2\nu )}$ is the bulk modulus of the poroelastic matrix.

According to Darcy’s law, the second term on the left-hand side in Equation (15) involves the fluid velocity, which is given by:

(18) $$v=\frac{-K}{\mu }(\nabla p+\rho_{f}g)$$

Here, g denotes the gravitational vector and is set to zero when gravity is neglected, μ represents the fluid viscosity. It should be noted that the permeability matrix K is obtained from Equation (13), and the permeability in the fracture zone is much higher than that in the intact zone.

Therefore, the final governing equation for fluid flow is:

(19) $$\rho_{f}S\frac{\partial p}{\partial t}+\nabla \cdot (-\frac{\rho_{f}K}{\mu }(\nabla p+\rho_{f}g))=Q_{m}-\rho_{f}\alpha \frac{\partial \epsilon_{vol}}{\partial t}$$

### 2.3. Interface Phase-Field Model

In this paper, the Voronoi tessellation method is employed for heterogeneous modeling of rocks. The interface phase-field model is introduced to simulate the weak mechanical properties at interfaces, which has been demonstrated by multiple studies to yield favorable simulation results [43,44,45].

Similar to the phase-field model, the interface phase-field model uses a scalar η to regularize the interface, where η=1 represents the interface, η=0 represents the mineral, and intermediate values η∈(0,1) represent a smooth transition. The diffuseness of the interface is controlled by the interface phase field length parameter $l_{i}$. The governing equation and boundary conditions for the interface phase field are:

(20) $$\eta -l_{i}^{2}\Delta \eta =0$$

(21) $$\left\{\begin{array}{l} \eta \left(x\right)=1\;\mathrm{on}\;x\in \Gamma_{i}\\ \nabla \eta \cdot n=0\;\mathrm{on}\;x\in \partial \Omega \end{array}\right.$$

Here, $\Gamma_{i}$ denotes the interface. By introducing the interface phase field η, the material parameters at the interface $\Theta_{i}$ and those inside the grain $\Theta_{g}$ can be smoothly blended as:

(22) $$\Theta_{s}\left(\eta \right)=\Theta_{i}\left[1-h\left(\eta \right)\right]+\Theta_{g}h\left(\eta \right),\;h\left(\eta \right)={\left(1-\eta \right)}^{2}$$

## 3. Numerical Experimental Program

### 3.1. Geometry and Boundary Conditions

**Figure 1:**
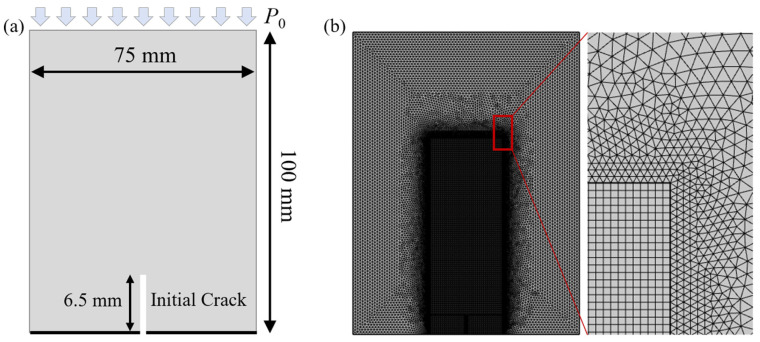
(**a**) Schematic of the specimen geometry; (**b**) illustration of the mesh configuration.

As shown in Figure 1a, the model is 100 mm in height and 75 mm in width. A single vertical initial crack is introduced at the center of the specimen, with a crack length of 6.5 mm. The mechanical boundary conditions are prescribed as follows: a fixed point and roller support is applied at the bottom boundary to eliminate rigid-body motion, and a uniaxial compressive load $P_{0}$ is imposed on the top boundary. The hydraulic boundary conditions are set as follows: the bottom boundary is impermeable, and the pore pressure on the top and lateral boundaries is fixed at 0. Fluid is injected into the pre-existing crack at a constant rate, denoted by $Q=1.67\times 10^{-6}\;m^{2}/s$.

According to the unified phase-field theory proposed by Wu [34], the dependence of simulations on $l_{0}$ is alleviated, allowing greater flexibility in its selection. In this study, $l_{0}$ is chosen by considering the model dimensions and the need to distinguish crack propagation modes at the grain scale. As $l_{0}$ approaches the grain size, distinguishing intergranular from transgranular cracking becomes increasingly difficult. Therefore, $l_{0}$ should be sufficiently small to resolve grains and grain boundaries, and reliable identification is generally achieved when $l_{0}$ does not exceed one-half of the equivalent grain diameter.

The interface phase field length parameter $l_{i}$ governs the transition from mineral grains to their boundaries. Because fractures in rocks do not strictly follow grain boundaries and the damaged zone is usually wider than the interface thickness, $l_{i}$ should be significantly smaller than $l_{0}$. Accordingly, $l_{0}$ is set to 0.1 mm and $l_{i}=0.2l_{0}$ in this study, as determined through repeated numerical calibration and verification. To compute the spatial distribution of mineral properties, the interface phase field variable is discretized with linear Lagrange elements over the entire computational domain. Along mineral boundaries, the maximum edge length of the free triangular elements is constrained to be no larger than $l_{i}$. A multiscale meshing strategy is adopted: the mesh is locally refined in the potential fracture propagation region, as shown in Figure 1b, where a mapped mesh with an element size of $0.25l_{0}$ is used; elsewhere, the extra fine free triangular mesh preset in COMSOL Multiphysics 6.0 is employed.

### 3.2. Material Parameters

In this study, the crystalline rock is idealized as a four-mineral aggregate consisting of quartz, albite, K-feldspar, and biotite, with volume fractions of 25%, 30%, 30%, and 15%, respectively. Both the mineral composition and the corresponding volume fractions were determined from X-ray diffraction (XRD) analyses of a large number of granite samples [46]. The mechanical parameters of each mineral are taken from nanoindentation tests [46] and the mechanical parameter table reported by Li et al. [47], as summarized in Table 1. The interfacial material parameters, except for $\nu_{0}$, are taken as one-half of those of the corresponding grains, while $\nu_{0}$ is kept unchanged. Fluid-related parameters are listed in Table 2.

Furthermore, this study mainly focuses on the effects of grain size and distribution on hydraulic fracturing. Although mineral grains in natural rocks are generally anisotropic and may affect the simulation results, this complexity is simplified in the present model. Therefore, grains of the same mineral phase are assumed to be homogeneous and isotropic.

### 3.3. Design of Numerical Experiments

In this study, rock heterogeneity is modeled using Voronoi polygon tessellation, in which the specimen is divided into irregular polygonal cells generated from a set of seed points. These cells are used to represent rock grains and internal boundaries. Its main advantage is that it provides a flexible and controllable description of rock mesostructure. By adjusting the seed distribution, the model can effectively control key variables such as grain size, grain shape, spatial distribution, mineral arrangement, and overall heterogeneity level. Therefore, Voronoi-based modeling has been widely used and proven effective in mesoscale and grain-scale studies of rock deformation and fracture [48,49,50,51].

Two groups of numerical experiments are conducted to investigate (i) mineral grain size and (ii) mineral distribution.

**Figure 2:**
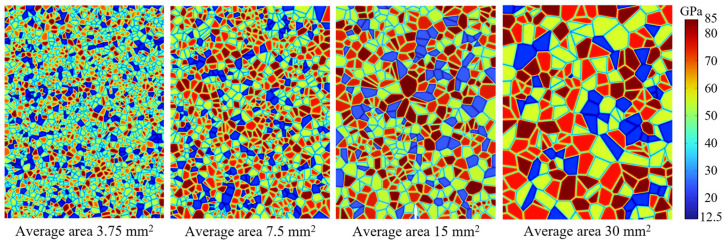
Spatial distribution of elastic modulus for models with different mean grain sizes.

**Figure 3:**
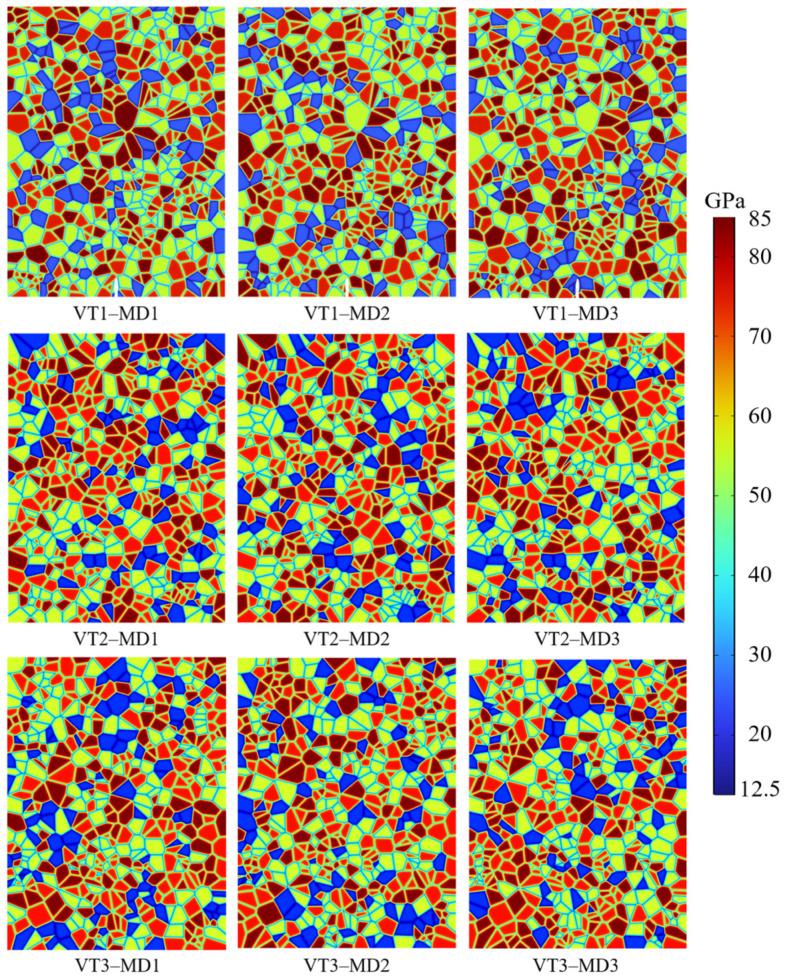
Spatial distribution of elastic modulus for different mineral distributions under different Voronoi tessellations.

(1) Grain size series: Grain size is characterized by the average grain area. As shown in Figure 2, Voronoi tessellations containing 250, 500, 1000, and 2000 grains are generated, corresponding to mean grain areas of 30, 15, 7.5, and 3.75 mm^2^, respectively. Four levels of axial stress are considered: 0, 0.01, 1, and 10 MPa.

(2) Mineral distribution series: Using Neper, three distinct Voronoi tessellation (VT) geometries with a mean grain area of mm^2^ are generated. For each VT geometry, three different mineral distributions (MDs) are constructed by permuting the Voronoi polygon IDs, as illustrated in Figure 3. Two axial stress levels are examined in this series: 0 MPa and 10 MPa.

Although the Voronoi tessellation method provides a computationally efficient and controllable framework for constructing synthetic rock microstructures, it remains an idealized simplification and cannot fully capture the irregular grain geometries and complex spatial features of natural rocks. To address this limitation, our team proposed the Expert K-Means Reconstruction Method (EKRM) [52], a modeling approach based on real rock microstructures, which introduces a probabilistic strategy for grain-boundary delineation and achieves higher reconstruction accuracy and reusability than existing methods. In future work, CT-based real rock structures will be incorporated and further coupled with the phase-field method to extend its application to fracture simulations in heterogeneous rocks.

### 3.4. Numerical Implementation and Validation

The hydro-mechanically coupled phase-field model involves multiple primary variables, including displacement, phase field, and pressure. To ensure numerical stability and improve the efficiency of the multiphysics coupling, the solution procedure is divided into three stages. First, the Voronoi-based heterogeneous material property fields are constructed by solving the interface phase field equation using the Helmholtz equation module. Second, a steady-state solution under the prescribed axial stress is computed and adopted as the initial condition for subsequent fluid injection into the pre-existing crack. Third, the transient fluid injection process and the accompanying hydraulically induced fracture evolution are solved, which constitutes the most computationally demanding part of the simulation.

For the multiphysics coupling, an alternate minimization algorithm is employed. Specifically, the displacement u and pressure p are first solved using an automatic Newton method, then the history variable H is updated. The phase field is then solved using the automatic Newton method. This staggered procedure is repeated until convergence. In the solver configuration, a second-order backward differentiation formula is adopted for time integration, together with Anderson acceleration to enhance convergence.

The validity of the present model has been verified through the single-edge notched plate shear test, the Sneddon analytical solution, and the Khristianovic-Geertsma-de Klerk (KGD) problem. Owing to space limitations, the details are not repeated here; more comprehensive information on the model validation and additional numerical examples can be found in our previous work [40].

## 4. Results

### 4.1. Crack Propagation Process

**Figure 4:**
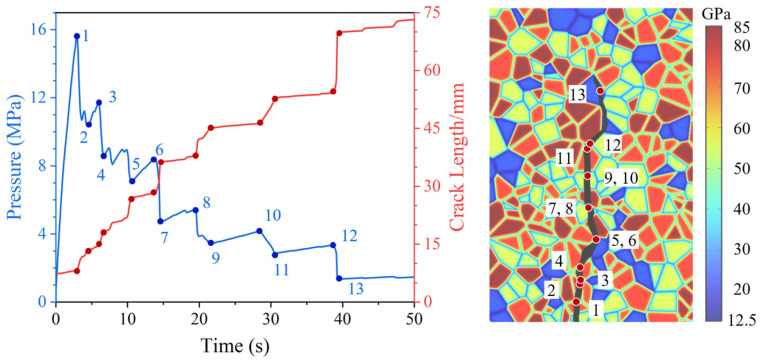
Hydraulic fracture propagation process: time histories of pressure and crack length with the evolving fracture path.

To illustrate the hydraulically induced crack growth process in heterogeneous rock, specimen VT3-MD1 under an axial stress of 10 MPa is selected, as shown in Figure 4. The results show a clear synchronization between the pressure and the crack length, and this coupled evolution is closely governed by microstructural heterogeneity. The propagation can be summarized as follows: when the fluid pressure reaches a critical level, the crack initiates and advances; once propagation occurs, fluid invades the newly created fracture volume, causing the pressure to drop until it can no longer drive further growth. With continued injection, the pressure gradually rebuilds and the fracture reopens; when the pressure again reaches a critical threshold, the crack continues to propagate. These features indicate that the proposed hydro-mechanically coupled phase-field fracture model captures the pressure variations associated with crack growth in a physically consistent manner.

Based on the evolution in Figure 4, the hydraulically induced crack growth can be categorized into two modes: rapid growth and stable growth. Rapid growth corresponds to segments such as 6–7 and 8–9 in Figure 4, whereas stable growth is represented by segments such as 5–6 and 9–10. Rapid growth typically occurs when the crack breaks through a relatively hard mineral grain and enters a softer mineral region. Because a higher energy threshold is required for propagation inside hard minerals, substantial energy accumulates in the specimen prior to breakthrough; after the crack penetrates the hard grain, the stored energy promotes a swift advance in the subsequent softer mineral. In contrast, stable growth often occurs when the crack propagates from a softer grain into a harder grain ahead, or when it bypasses an upcoming grain through a large angle deflection. In such cases, the fracture transitions from rapid advance within the soft mineral to a temporary arrest in front of the next harder grain. Because penetrating through or detouring around this hard mineral requires a higher driving pressure, a distinct period of pressure build-up is observed prior to the subsequent rapid advance. During this pressure-rising stage, intergranular cracking that bypasses the hard grain may occur, and the fracture can advance stably over a short distance (e.g., 11–12). In contrast, when the obstacle ahead is a hard grain (e.g., quartz) and the potential path involves transgranular cracking, little or no crack extension is observed during the pressure build-up stage (e.g., 5–6 and 7–8). These observations underscore the sensitivity of hydraulic fracture growth to local mineral arrangements, motivating further analysis of grain size and distribution effects.

### 4.2. Evolution of Hydraulically Induced Crack Growth Under Different Grain Sizes

**Figure 5:**
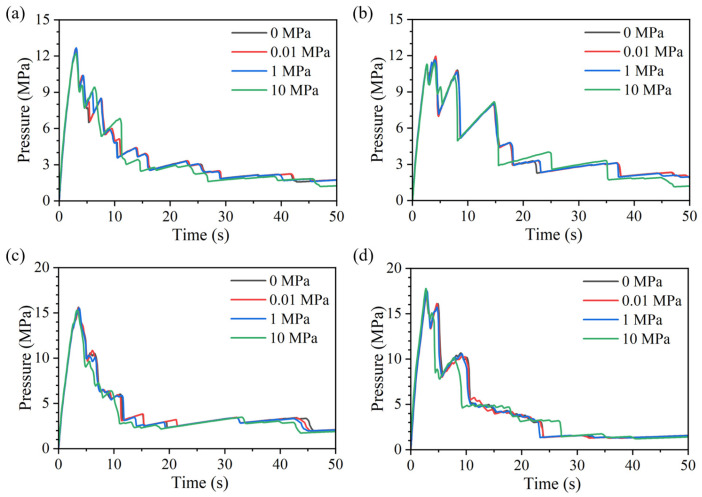
Pressure-time curves under different axial stresses for models with mean grain areas of (**a**) 3.75 mm^2^, (**b**) 7.5 mm^2^, (**c**) 15 mm^2^, and (**d**) 30 mm^2^.

With continued fluid injection, the pressure within the crack evolves continuously. The simulation results indicate that, under different mineral grain size conditions, the pressure-time curves exhibit an overall consistent evolution pattern (Figure 5).

**Figure 6:**
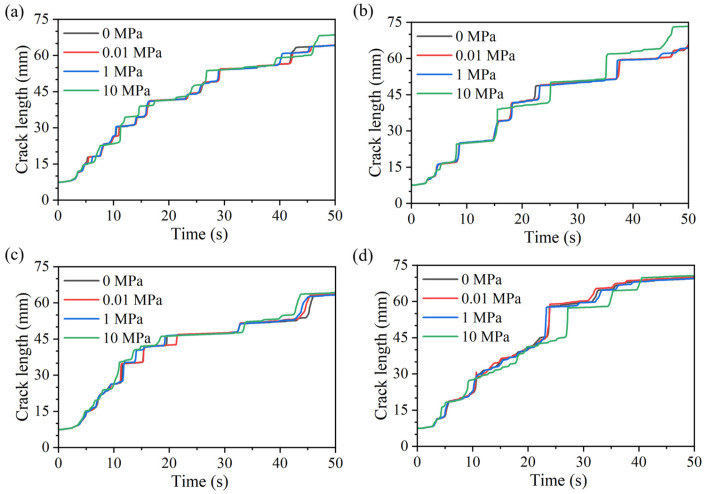
Crack length-time curves under different axial stresses for models with mean grain areas of (**a**) 3.75 mm^2^, (**b**) 7.5 mm^2^, (**c**) 15 mm^2^, and (**d**) 30 mm^2^.

During hydraulically driven fracture propagation, the injection pressure first increases in an approximately linear manner. As damage accumulates near the crack tip and a seepage pathway gradually develops, the slope of the curve decreases slightly. After the pressure reaches a peak, crack initiation occurs and the fracture begins to propagate forward. The evolution of fracture length with time is shown in Figure 6, and a clear correspondence between pressure and fracture growth is observed, consistent with the trends described above. When the fracture enters a relatively soft mineral, it propagates more readily, manifested by a sudden increase in fracture length accompanied by a pronounced pressure drop. In contrast, when the fracture approaches a harder mineral region, propagation slows markedly or even temporarily arrests; crack length growth becomes delayed, while the continuously injected fluid accumulates and the pressure gradually rises until it again reaches a critical threshold that triggers a new episode of crack advance and pressure release. Owing to the heterogeneous grain distribution, such fluctuations recur throughout the propagation process.

In most cases, the pressure peak coincides with the first pronounced pressure drop. However, depending on the local mineral arrangement, the response may exhibit a small pressure drop followed by renewed pressure build-up to the overall peak, as in Figure 5b, again highlighting the role of heterogeneity. In this case, the region ahead of the initial crack tip is first occupied by a relatively weak biotite grain; the fracture can traverse this grain more easily, leading to a transient pressure reduction. It then encounters a stronger mineral region, where propagation is impeded, causing pressure to re-accumulate and eventually reach the global peak. Because mineral distribution can substantially alter the pressure response, the peak pressure shows no clear positive or negative correlation with grain size.

**Figure 7:**
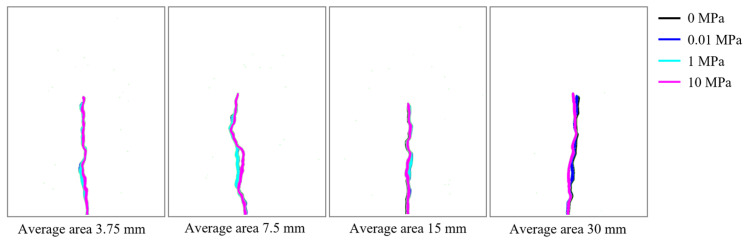
Crack propagation paths for grain-size models under all loading cases.

Comparing the results at the same grain size but different axial stresses in Figure 5 and Figure 6 indicates that the influence of axial stress on the pressure and crack-length evolution is not pronounced. In particular, before crack propagation occurs, the pressure and crack-length curves under different axial stresses almost fully overlap; noticeable differences emerge mainly after the peak pressure, when the crack enters the rapid-growth stage. Even then, the overall evolution patterns remain similar, suggesting that the effect of axial stress on the crack-growth behavior is relatively limited in magnitude. The fracture morphologies for different axial stresses and grain-size models are summarized in Figure 7, where results are overlaid with higher axial-stress cases plotted on top and distinguished by color. As shown in Figure 7, axial stress mainly introduces local differences in the propagation path, while the overall macroscopic trend remains broadly consistent.

Collectively, these results indicate that, under different axial stresses, the pressure evolution and crack-growth trends are highly similar, and the dominant controlling factor for the crack path and its evolution is still the mineral distribution. Thus, when the mineral distribution is identical, the temporal evolution of fracture pressure and crack length remains largely consistent even under different axial stresses.

**Figure 8:**
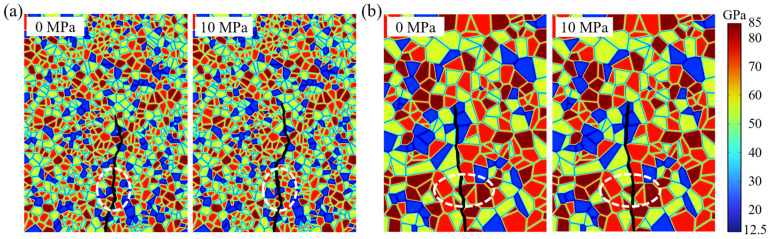
Effect of axial stress on hydraulically induced crack paths: (**a**) mean grain area 7.5 mm^2^; (**b**) mean grain area 30 mm^2^.

For specimens in which the crack path is more evidently affected by axial stress, typical differences are observed in the model with a mean grain area of 7.5 mm^2^, as shown in Figure 8. Under 10 MPa axial stress, the crack in the white-circled region penetrates a quartz grain directly, forming a distinct transgranular fracture; by contrast, under 0–1 MPa axial stress, the crack at the same location primarily propagates along the quartz-grain boundary, exhibiting intergranular fracture. A similar behavior is observed for the model with a mean grain area of 30 mm^2^, where transgranular cracking becomes more pronounced under 10 MPa. These observations indicate that higher axial stress fundamentally changes the fracture mechanism from intergranular to transgranular in specific zones. Specifically, higher axial stress makes transgranular cracking more likely to occur in high-strength minerals such as quartz.

### 4.3. Evolution of Hydraulically Induced Crack Growth Under Different Mineral Distributions

**Figure 9:**
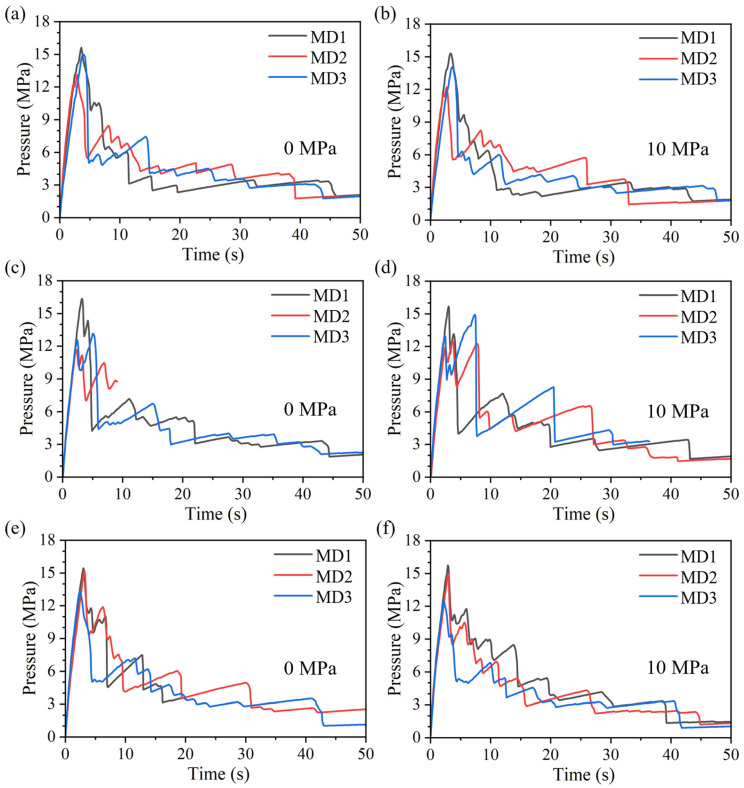
Pressure-time curves for different mineral distributions: (**a**,**b**), (**c**,**d**), and (**e**,**f**) correspond to Voronoi tessellations 1, 2, and 3, respectively.

**Figure 10:**
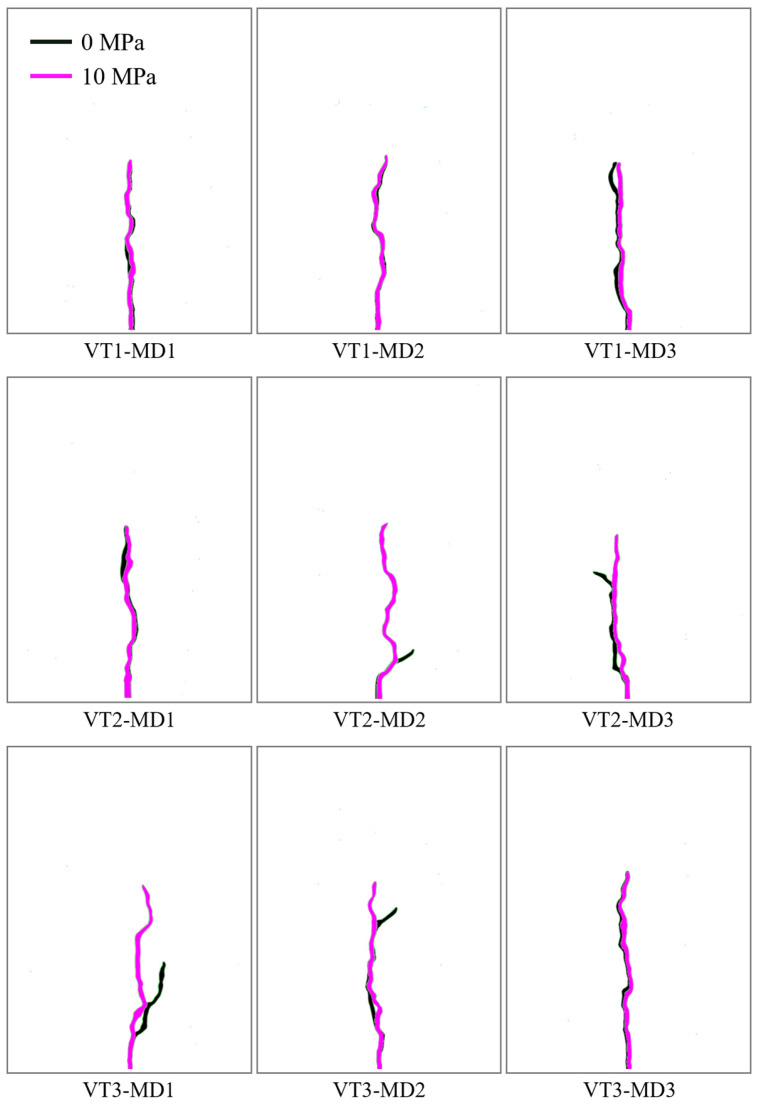
Crack propagation paths for models with different mineral distributions.

The pressure-time curves for different mineral distributions are shown in Figure 9, and the corresponding crack paths are presented in Figure 10. Overall, the basic evolution pattern is consistent with that described above: the pressure rises rapidly to a peak at the early injection stage; after the peak, the system enters a rapid crack-growth stage, during which heterogeneity induces local pressure oscillations characterized by repeated rises and drops. The detailed process is not repeated here.

Although the overall trends are similar, mineral distribution exerts a significant influence on the pressure response and crack-growth outcomes. Under an identical polygonal mesh geometry, changing only the mineral distribution can still cause substantial differences in the pressure response and propagation results. In conjunction with the crack paths, the influence of mineral distribution under the same tessellation can be summarized in two scenarios: (1) the mineral distribution alters the macroscopic propagation trajectory; (2) the macroscopic path remains broadly similar. Regardless of the scenario, however, the mineral assemblage in the vicinity of the evolving crack path can differ markedly, leading to different local resistances experienced by the crack tip and thus different pressure evolutions.

In addition, comparison of pressure curves under different axial stresses indicates that, for some cases (e.g., VT2-MD2), the simulation under 0 MPa axial stress could not be continued to 50 s. This occurs because the crack path deviates substantially from the vertical direction, reaches the boundary of the mesh-refinement zone earlier, and thereby triggers premature termination of the computation.

**Figure 11:**
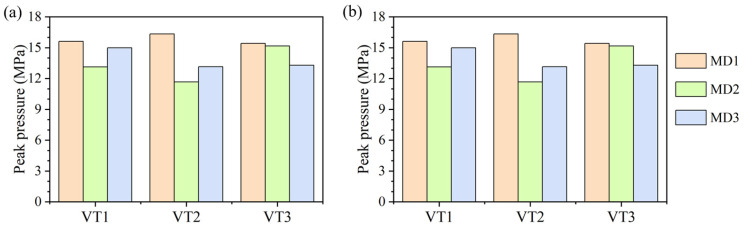
Peak pressures for different mineral distributions under two axial stress levels: (**a**) 0 MPa; (**b**) 10 MPa.

To clarify the factors controlling the peak pressure, the statistical results for all cases in Section 4.3 with different mineral distributions under different axial stresses are summarized in Figure 11. The results indicate that axial stress is not the dominant factor: when the axial stress increases from 0 to 10 MPa, the peak pressure changes only slightly. A paired-sample *t*-test was therefore conducted to further evaluate the effect of axial stress, and the result (*p* = 0.740) confirms that the difference is not statistically significant at the 0.05 level. In contrast, even under the same axial stress and Voronoi tessellation, considerable scatter is observed among specimens with different mineral arrangements, with the peak-pressure difference exceeding 4 MPa, suggesting that mineral distribution is the primary factor controlling peak-pressure variability. For the 18 specimens considered, the mean peak pressure and standard deviation are 14.31 MPa and 1.78 MPa at 0 MPa axial stress, and 14.21 MPa and 1.74 MPa at 10 MPa axial stress, respectively. Because all specimens share identical mineral fractions and the same mineral properties, the dispersion of peak pressure mainly originates from microstructural heterogeneity, namely the modulation of local propagation resistance at the crack tip induced by differences in mineral distribution.

**Figure 12:**
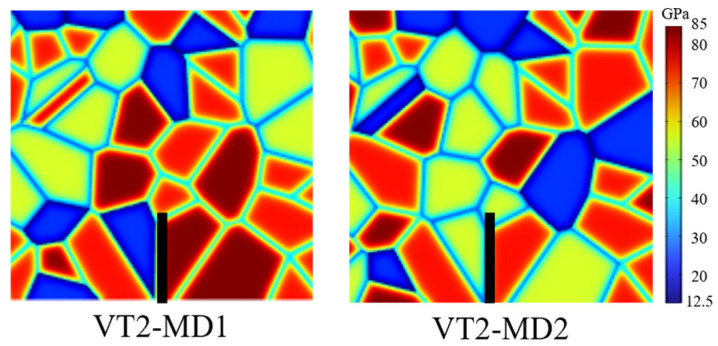
Representative mineral-grain configurations near the initial crack tip.

A specimen-by-specimen examination of mineral configurations near the initial crack tip further reveals that higher peak pressures typically occur when strong minerals such as quartz or albite occupy the region ahead of the crack tip, thereby increasing the resistance to crack initiation and early growth; conversely, when relatively weak minerals dominate the crack-tip neighborhood, the peak pressure is lower. A representative contrast is observed for VT2: MD1 yields the highest peak pressure, whereas MD2 yields the lowest. As shown in Figure 12, the crack-tip neighborhood in MD1 is dominated by quartz, while that in MD2 is dominated by K-feldspar, leading to a pronounced divergence in peak pressure and further underscoring the controlling role of mineral distribution.

**Figure 13:**
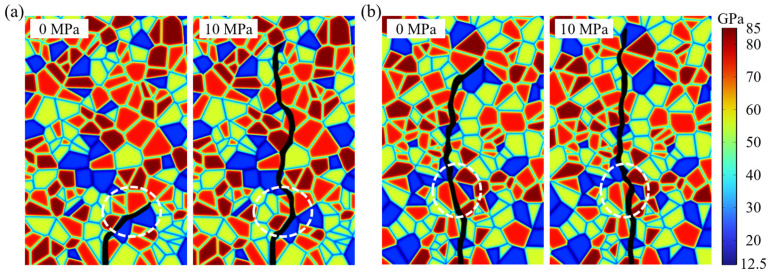
Crack paths under axial stresses of 0 MPa and 10 MPa: (**a**) VT2-MD2; (**b**) VT3-MD2.

Consistent with the observations in Section 4.2, higher axial stress fundamentally changes the fracture mechanism from intergranular to transgranular in specific zones during crack propagation. As shown in Figure 13a for VT2-MD2, within the region marked by the white dashed box, the crack under 0 MPa axial stress propagates mainly along biotite boundaries; when the axial stress increases to 10 MPa, the crack no longer continues along the biotite boundaries, but instead advances along the albite boundary and turns toward the vertical direction. This behavior can be interpreted using an energy minimization principle: under 10 MPa axial stress, the candidate path that would continue along the biotite boundary is no longer the energetically preferred evolution, and the crack therefore selects a new direction that reduces the total system energy. A similar phenomenon is observed for VT3-MD2, as shown in Figure 13b, where a crack that would otherwise follow the albite boundary under low axial stress becomes a transgranular fracture through the albite grain under 10 MPa. From an energy perspective, although transgranular fracture through albite requires a higher fracture energy expenditure, if the crack instead continues to propagate along the albite grain boundary, the trajectory would remain markedly deviated from the vertical direction, thereby producing a larger increase in elastic free energy after advance. When the competition between surface energy and elastic free energy is accounted for, transgranular fracture can become the more favorable route for minimizing the total energy, and is therefore selected.

It should be emphasized that the tendency for the crack to propagate toward the vertical direction is fundamentally dictated by the macroscopic stress state. In principle, hydraulically induced fractures tend to propagate perpendicular to the maximum principal stress; in the homogeneous model, the crack path typically remains stably vertical. In heterogeneous media, local mineral distributions can cause local deflections or mode transitions, while increased axial stress reshapes the energy competition and promotes a return toward the dominant propagation direction imposed by the macroscopic stress field.

## 5. Discussion

**Figure 14:**
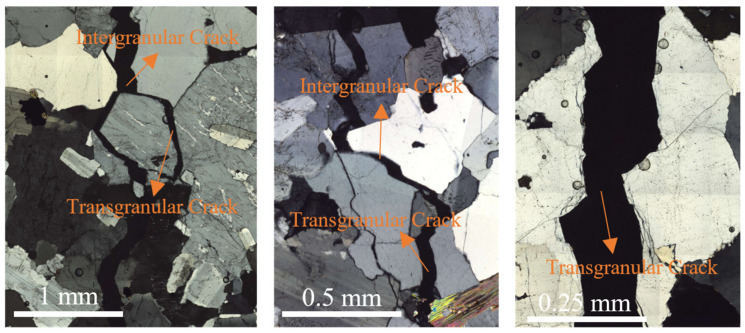
Experimental observations of intergranular and transgranular fracture propagation in thin rock sections during hydraulic fracturing.

Although this study is based purely on numerical simulations, the simulated fracture patterns are in good agreement with experimental observations, indicating that the proposed model can reasonably capture the controlling effect of rock microstructure on hydraulic fracture propagation. In this study, phase field simulations of hydraulic fracturing revealed the widespread occurrence of both intergranular and transgranular cracks. This phenomenon was also observed in our hydraulic fracture propagation experiments on thin rock sections with visually identifiable grain structures, as shown in Figure 14. It can be seen that mineral grains are closely associated with crack evolution and play an important role in controlling crack path selection. In general, fractures tend to propagate along the boundaries of mineral grains, indicating a preference for intergranular cracking. However, when a mineral grain is relatively large and propagation along the grain boundary would require a significant detour, the fracture is more likely to cut through the grain, resulting in transgranular cracking.

Additionally, the results of this study are in good agreement with previous experimental findings. Based on triaxial hydraulic fracturing experiments combined with micro-CT observations, Zhang et al. [53] reported results consistent with those presented in Section 4.1, showing that cracks tend to propagate along grain boundaries rather than through mineral grains. Similarly, from triaxial hydraulic fracturing experiments with CT observations, Zhuang et al. [54] obtained findings consistent with those in Section 4.2 and Section 4.3, indicating that mineral grains are closely associated with crack evolution and play an important role in controlling crack path selection. Preferred mineral orientation, relative to the maximum principal stress, governs the geometry of hydraulic fracture propagation: when it is parallel to the maximum principal stress, the fracture propagates along the foliation; when it is not parallel, the fracture is deflected by weak planes and follows a more tortuous path.

## 6. Conclusions

This study employs a hydro-mechanical phase-field model with Voronoi-based microstructures to simulate hydraulic fracturing in crystalline rock containing a single initial crack. By varying grain size and mineral distribution under different axial-stress levels, we quantify how heterogeneity controls the coupled evolution of injection pressure, crack length, and fracture trajectory. The main conclusions are as follows:

1. Injection pressure evolution is closely correlated with stepwise crack growth, governed by rock heterogeneity. Rapid advances occur in weak mineral regions, while propagation is impeded in strong minerals, requiring continued pressurization until a threshold is reached.
2. Mineral distribution is the primary factor influencing peak pressure and fracture evolution, whereas axial stress exerts a minor effect. The mean peak pressures under 0 MPa and 10 MPa are similar (14.31 vs. 14.21 MPa), yet altering mineral distribution alone can cause peak pressure differences exceeding 4 MPa. Higher peak pressures occur when strong minerals are positioned ahead of the crack tip, increasing initiation resistance.
3. Under low axial stress, fractures preferentially propagate along mineral boundaries, while higher axial stress fundamentally changes the fracture mechanism from intergranular to transgranular in specific zones. This behavior aligns with energy minimization principles, as high axial stress increases the elastic free energy penalty for interface detouring, favoring paths consistent with the macroscopic stress field.

## Figures and Tables

**Table 1:** Mechanical parameters of different minerals in crystalline rock [43].

Material Type (Mineral)	$E_{0}$/GPa	$f_{t}$/MPa	$\nu_{0}$	$G_{c}$/N·m^−1^
Quartz	85	30	0.15	55
K-feldspar	55	20	0.2	35
Albite	75	25	0.2	45
Biotite	25	10	0.3	15

**Table 2:** Fluid-related parameter values used in the model.

Parameters	Values	Parameters	Values
The porosity of the matrix ($\varepsilon_{pR}$)	5 × 10^−3^	Permeability of reservoir domain ($k_{R}$)	5 × 10^−15^ m^2^
The porosity of the fracture ($\varepsilon_{pF}$)	1	Permeability of fractured domain ($k_{F}$)	1 × 10^−8^ m^2^
Fractured zone threshold ($\phi_{c1}$)	0.98	Biot’s coefficient of the matrix ($\alpha_{R}$)	0.3
Transition zone threshold ($\phi_{c2}$)	0.4	Biot’s coefficient of the fracture ($\alpha_{F}$)	1
Fluid dynamic viscosity (μ)	1 × 10^−3^ Pa·s	Fluid compressibility (c)	5.1 × 10^−12^ 1/Pa

## Data Availability

The original contributions presented in this study are included in the article. Further inquiries can be directed to the corresponding author.
